# Spitting of Blood

**Published:** 1895-08

**Authors:** 


### SPITTING OF BLOOD.

Dr. Jas. D. Morgan, of Washington, D. C., in the *Virginia Medical Monthly,* in discoursing upon the need of care in the diagnosis of spitting of blood, says :

Several cases of epistaxis and spitting of blood have come under my care where the hæmorrhage was but a precursor of a delayed resolution in pneumonia. The lungs and stomach I have often seen the outlocks of a vicarious menstruation, and I may state here without resultant bad effect. So it is important not only to locate the hæmorrhage, but more important to establish the cause. A hæmorrhage from the lung is most apt to be considered correlative of phthisis. Even Flint has said that " in a certain proportion of cases it occurs when physical signs do not afford evidence of its existence." A bronchorrhagia may depend solely on a mitral or aortic lesion ; "a sputa streaked with blood or simple bronchitis, or intimately admixed with blood on pneumonia ;" a pulmonary hæmorrhage may be symptomatic of delayed menstruation or impede portal circulation. Sir Andrew Clark speaks of a " non-tubercular hæmoptysis in elderly persons due to structural alterations in the blood-vessels of the lungs in persons of the arthritic diathesis. The hæmorrhage is aggravated or maintained by the administration of astringents." A sudden and great loss of blood may come from a ruptured aneurism into the bronchus, or from an ulceration of some portion of the respiratory tract extending into the adjacent artery, as has been reported of "the larnyx into the carotid artery." Slight spitting of blood may be due to a blow on the chest, or to a mechanical injury, such as a fractured rib; or the cause of the hæmorrhage may not be apparent, as occurs sometimes in the beginning or in the latent forms of certain contagious diseases.

As to hæmorrhage from other parts, as the mouth or nasopharynx, direct inspection will always reveal the source.

Many of the following points in the differential diagnosis of hæmatemesis and hæmoptysis are to be found if sought for industriously:

| HÆMATEMESIS. | HÆMOPTYSIS. |
|---|---|
| Usually antecedent history of gastric or hepatic disease or portal congestion. | Usually antecedent history of lung or heart disease. |
| Preceded by nausea and vomiting. | Preceded by dyspnœa, cough, salty taste; warm feeling over sternum, sense of trickling of fluid in chest, and generally followed by nausea and vomiting. Moist râles on auscultation. |
| Blood acid, dark grumous, generally more abundant; most likely mixed with food. Tenderness over stomach. Generally blood with stools. | Blood alkaline; bright frothy red. Subsequent cough, with mucus tinged with blood. |

These points and others passing rapidly before the mind are but the resources of a thorough diagnostician, and are the footings on which a prompt and successful treatment is laid down.

It is only the *tactus eruditus*, the acoustic ear, the quick, comprehensive, and discriminating eye which can lead us to the adoption of a ready and safe treatment for the spitting of blood.—*Medical Examiner*.

